# HiCLift: a fast and efficient tool for converting chromatin interaction data between genome assemblies

**DOI:** 10.1093/bioinformatics/btad389

**Published:** 2023-06-19

**Authors:** Xiaotao Wang, Feng Yue

**Affiliations:** Department of Biochemistry and Molecular Genetics, Feinberg School of Medicine, Northwestern University, Chicago, IL 60611, United States; Department of Biochemistry and Molecular Genetics, Feinberg School of Medicine, Northwestern University, Chicago, IL 60611, United States; Robert H. Lurie Comprehensive Cancer Center of Northwestern University, Chicago, IL 60611, United States

## Abstract

**Motivation:**

With the continuous effort to improve the quality of human reference genome and the generation of more and more personal genomes, the conversion of genomic coordinates between genome assemblies is critical in many integrative and comparative studies. While tools have been developed for such task for linear genome signals such as ChIP-Seq, no tool exists to convert genome assemblies for chromatin interaction data, despite the importance of three-dimensional genome organization in gene regulation and disease.

**Results:**

Here, we present HiCLift, a fast and efficient tool that can convert the genomic coordinates of chromatin contacts such as Hi-C and Micro-C from one assembly to another, including the latest T2T-CHM13 genome. Comparing with the strategy of directly remapping raw reads to a different genome, HiCLift runs on average 42 times faster (hours vs. days), while outputs nearly identical contact matrices. More importantly, as HiCLift does not need to remap the raw reads, it can directly convert human patient sample data, where the raw sequencing reads are sometimes hard to acquire or not available.

**Availability and implementation:**

HiCLift is publicly available at https://github.com/XiaoTaoWang/HiCLift.

## 1 Introduction

The reference genome is essential for genomic research, as it provides a standardized coordinate system for mapping high-throughput sequencing reads and annotating genomic elements. However, the reference genome of different species has undergone numerous modifications over the years. Hundreds of thousands of genomic studies have been performed and the sequencing data have been analyzed against different versions of reference genome. To facilitate an integrative analysis of the published data, it would be necessary to convert the different sequencing data into a consistent coordinate system. In general, two approaches can be considered. The first approach is to remap the original sequencing reads to the same target assembly. This approach provides the most accurate result but is computationally intensive and time-consuming. The second approach is to convert the genomic coordinates between assemblies by using a mapping file. Although there can be information loss during the conversion, this approach gives a good trade-off between performance and accuracy for most applications. Several tools have been developed to perform the coordinate conversion for datasets coming from various experiments (Kuhn *et al.* 2013, Zhao *et al.* 2014). However, despite the importance of three-dimensional (3D) genome organization in gene regulation and disease, no tool exists to convert genome assemblies for chromatin interaction data.

Recent years have seen a growth spurt of 3D genome technologies, such as Hi-C (Lieberman-Aiden *et al.* 2009), Micro-C (Hsieh *et al.* 2015), ChIA-PET (Fullwood *et al.* 2009), HiChIP (Mumbach *et al.* 2016), DNA SPRITE (Quinodoz *et al.* 2018), and ChIA-Drop (Zheng *et al.* 2019). There have been over 600 datasets generated to study the higher-order chromatin structure and the interactions between genes and their distal regulatory elements (Reiff *et al.* 2022). In general, each experiment has several hundred million or billions of raw reads. Due to deep sequencing, reprocessing these data to a specific genome assembly could be extremely time-consuming. More importantly, there have been many Hi-C datasets that were generated for human individuals or patient samples, and the raw sequencing reads are not publicly available, which severely hinder our ability to integrate such data with the newly generated data.

Here, we introduce HiCLift (Supplementary Fig. S1), an efficient command-line tool that can convert genomic coordinates of chromatin contacts between assemblies. HiCLift supports a variety of data formats that are widely used by the 3D genome community. Using large Hi-C datasets as a benchmark, we show that compared with the strategy directly remapping raw reads to a different genome, HiCLift runs on average ∼42 times faster, while outputs nearly identical contact matrices.

## 2 Results

### 2.1 HiCLift accurately converts genomic coordinates of chromatin contacts from one assembly to another

We benchmarked the performance of HiCLift using three Hi-C datasets of different species, including a human dataset IMR90 (∼1.54 billion reads), a mouse dataset CH12-LX (∼1.38 billion reads), and a zebrafish muscle dataset (∼1.35 billion reads). For each dataset, we used the contact maps derived from read remapping as a reference to evaluate the accuracy of HiCLift.

We first compared the overall distribution of chromatin contacts at 50 kb resolution using the stratum-adjusted correlation coefficients (SCCs) (Yang *et al.* 2017). The SCCs between HiCLift and read remapping achieved to 0.9987 (Supplementary Fig. S2), 0.9997 (Supplementary Fig. S3), and 0.9883 (Supplementary Fig. S4) for the IMR90, CH12-LX, and zebrafish muscle datasets, respectively, suggesting that the coordinates converted from other genome assembly versions by HiCLift are highly concordant with the coordinates from read remapping.

**Figure 1.**
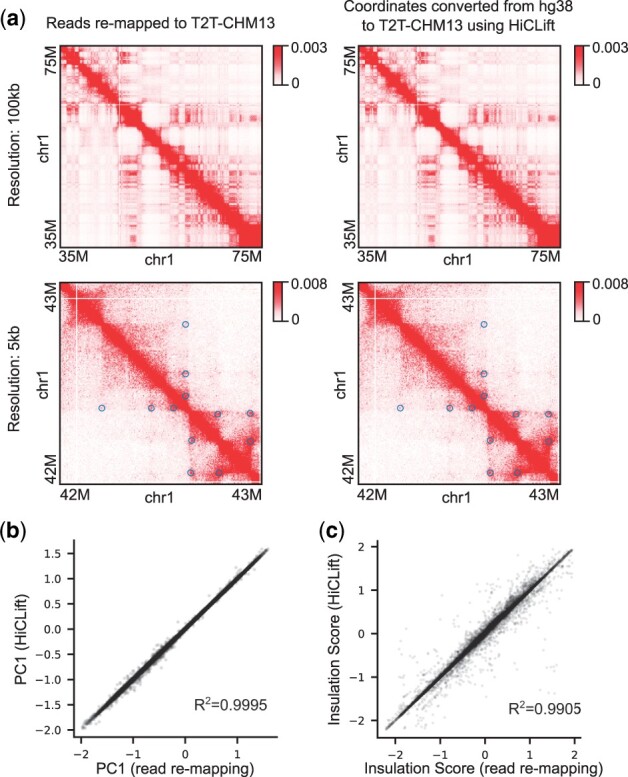
Accuracy of HiCLift on the IMR90 Hi-C dataset. (a) Example regions comparing contact matrices obtained from HiCLift (second row) with the matrices we generated by remapping the raw reads (first row). The blue circles in the second column indicate the detected chromatin loops on corresponding maps. (b) The first principal component (PC1) for characterizing the chromatin compartment pattern at 100 kb resolution is compared between the two methods. (c) The insulation scores for capturing chromatin domain boundaries at 25 kb resolution are compared between the two methods.

Next, we evaluated the concordance of HiCLift and read remapping in detecting specific contact patterns, such as those induced by chromatin compartments, topologically associating domains (TADs), chromatin loops, and interchromosomal translocations (Wang *et al.* 2022) (Fig. 1 and Supplementary Figs S5 and S6). Both chromatin compartments (measured by PC1) and TADs (measured by insulation scores) called from HiCLift and read remapping gave highly similar results, achieving coefficients of determination ($R^{2}$) of 0.9995 and 0.9905, respectively, for the IMR90 dataset, 1.0000 and 0.9957 for the CH12-LX dataset, and 0.9995 and 0.9635 for the zebrafish muscle dataset. Chromatin loops were also highly concordant between HiCLift and read remapping, with overlap coefficients of 0.9444, 0.9925, and 0.9514 for IMR90, CH12-LX, and zebrafish muscle, respectively. As for the interchromosomal translocations, we focused on CH12-LX, where a known translocation occurs between chromosomes chr7 and chr17 (Supplementary Fig. S7). We observed nearly identical interactions between HiCLift and read remapping around the breakpoints of this translocation, suggesting the accuracy of HiCLift in converting interchromosomal contacts.

In light of the fact that certain inaccurately assembled regions in older reference genome can be corrected using chain files that map to newer versions, we anticipate that HiCLift can enhance the contact matrices within these regions. To verify this, we focused on the human H19/ICF2 locus. In hg38, the LINC01150 gene at this locus has been demonstrated to be inverted and translocated upstream of the TNNT3 gene, as opposed to the most recent T2T-CHM13 genome (Battaglia *et al.* 2022, Nurk *et al.* 2022) (Supplementary Fig. S8a). Accordingly, when we mapped reads from a Micro-C dataset to hg38, we observed abnormal interaction blocks between LINC01150 and TNNT3. However, when the reads were mapped to T2T-CHM13, no such blocks were observed (Supplementary Fig. S8b). Consistent with our previous evaluations, utilizing HiCLift to lift over contact coordinates from hg38 to T2T-CHM13 produced nearly identical contact matrices to those obtained by directly mapping reads to T2T-CHM13.

In addition to contact pairs at the base-pair resolution, we also evaluated the accuracy of HiCLift when contact matrices binned at coarser resolutions are used as input. As expected, the SCCs between HiCLift and read remapping decreased as the bin sizes of the input matrix increased (Supplementary Figs S2–S4). However, even with the 10 kb contact matrices as input, the SCCs still achieved 0.9986, 0.9992, and 0.9853 for the IMR90, CH12-LX, and zebrafish muscle datasets, respectively.

Together, these analyses show that HiCLift can accurately convert genomic coordinates of chromatin contacts between different genome assemblies for various species.

### 2.2 HiCLift runs 42 times faster than read remapping

As 3D genome libraries usually need deep sequencing for effective downstream analysis, processing these data from scratch is often time-consuming. For example, processing the benchmark datasets in this study from raw sequencing reads to sorted 4DN pairs took ∼52.8, 45.2, and 84.9 h for IMR90, CH12-LX, and zebrafish muscle, respectively (8 CPU cores were allocated). As a comparison, HiCLift took ∼2.2 (24×), 1.4 (32×), and 1.2 h (71×) to finish the coordinate conversion, which was on average 42 times faster than a standard Hi-C data processing pipeline (Supplementary Fig. S9a). To test the performance of HiCLift at various sequencing depths, we computationally down-sampled the IMR90 dataset into nine different depths (ranging from 100 million to 900 million contact pairs). Overall, the running time for HiCLift grew linearly with the sequencing depths, while the memory usage remains constant (Supplementary Fig. S9b and c).

## 3 Discussion

In this work, we developed a novel computational tool HiCLift and demonstrated its accuracy and efficiency for converting genomic coordinates of chromatin contacts between assemblies. Although one may apply the tool with arbitrary chain files, like other liftover tools, HiCLift was optimized only for intraspecies genomic conversions. As the volume of chromatin interaction data keeps increasing, we envision HiCLift to play a vital role in an integrative or comparative analysis when remapping raw reads is not feasible.

## Supplementary Material

btad389_Supplementary_Data

## Data Availability

The IMR90 and the CH12-LX Hi-C datasets were downloaded from the GEO database with accession code GSE63525. The zebrafish muscle dataset was downloaded from GEO with accession code GSE134055. And the Micro-C dataset was downloaded from GEO with accession code GSE163666.
