# Supplementary material for: HiCLift: a fast and efficient tool for converting chromatin interaction data between genome assemblies
Source: Bioinformatics. 2023 Jun 19;39(6):btad389. doi: 10.1093/bioinformatics/btad389 (PMC10313346; doi:10.1093/bioinformatics/btad389)
Supplement: btad389_Supplementary_Data [file btad389_supplementary_data.pdf]

HiCLift: A fast and efficient tool for converting chromatin interaction data between genome assemblies

Xiaotao Wang[1,2,3#], Feng Yue[3,4#]

[1] Obstetrics and Gynecology Hospital, Institute of Reproduction and Development, Fudan University, Shanghai, China.

[2] Research Units of Embryo Original Diseases, Chinese Academy of Medical Sciences, Shanghai, China.

[3] Department of Biochemistry and Molecular Genetics, Feinberg School of Medicine Northwestern University, Chicago, Illinois, USA.

[4] Robert H. Lurie Comprehensive Cancer Center of Northwestern University, Chicago, Illinois, USA.

[#] Correspondence: Yue@northwestern.edu, wangxiaotao@fudan.edu.cn

Supplementary Method S1. Hi-C data sources and processing

Supplementary Method S2. ChIP-Seq data sources and processing

Supplementary Method S3. Overview of the HiCLift framework

Supplementary Method S4. Coordinate conversion for contact pairs

Supplementary Method S5. Coordinate conversion for contact matrices

Supplementary Method S6. Description and source of chain files

Supplementary Method S7. Construction of interval trees from a chain file

Supplementary Figure S1. The overall design of HiCLift.

Supplementary Figure S2. Comparison of Hi-C contact maps at 50kb resolution derived from read re-mapping and HiCLift on the IMR90 dataset

Supplementary Figure S3. Comparison of Hi-C contact maps at 50kb resolution derived from read remapping and HiCLift on the CH12-LX dataset

Supplementary Figure S4. Comparison of Hi-C contact maps at 50kb resolution derived from read remapping and HiCLift on the zebrafish dataset

Supplementary Figure S5. Accuracy of HiCLift on a mouse Hi-C dataset

Supplementary Figure S6. Accuracy of HiCLift on a zebrafish Hi-C dataset

Supplementary Figure S7. Accuracy of HiCLift in converting inter-chromosomal contacts in CH12-LX

Supplementary Figure S8. Accuracy of HiCLift at inaccurately assembled regions.

Supplementary Figure S9. Computational efficiency of HiCLift on the benchmark datasets

Supplementary Method S1. Hi-C data sources and processing

The Hi-C datasets of a human fibroblast cell line IMR90 and a mouse lymphoma cell line CH12-LX were downloaded from the GEO database with accession code GSE63525 (Rao, et al., 2014). The Hi-C dataset of a zebrafish muscle tissue was downloaded from GEO with accession code GSE134055 (Yang, et al., 2020). The Micro-C dataset of H1-ESC cells were downloaded from GEO with accession code GSE163666 (Akgol Oksuz, et al., 2021). The raw sequencing reads were processed using runHiC (https://pypi.org/project/runHiC/, v0.8.6), a command-line tool based on the 4D Nucleome Hi-C data processing pipeline (https://data.4dnucleome.org/resources/data-analysis/hi_c-processing-pipeline). To benchmark the performance of HiCLift, each dataset was first processed into two versions of chromatin contacts using two different genome assemblies, and then the coordinates of chromatin contacts mapped to the older genome version were converted into the newer genome version using HiCLift, and compared with chromatin contacts directly mapped to the newer genome version. Specifically, the IMR90 dataset was processed into hg38 and T2T-CHM13 (v2.0), the CH12-LX dataset was processed into mm10 and mm39, and the zebrafish muscle dataset was processed into danRer10 and danRer11.

We compared the contact maps derived from runHiC and HiCLift at different scales. First, we measured the overall similarity between two contact maps by using the stratum-adjusted correlation coefficients (SCC) at the 50kb resolution (Yang, et al., 2017). Second, we compared the chromatin compartments measured by the first eigenvector (PC1) of the normalized contact matrices at 100kb. Third, we compared the domain boundary strength measured by the insulation scores at 25kb. Finally, we compared the identified chromatin loops at the 5kb resolution.

Specifically, SCCs were computed using a Python implementation of the original HiCRep algorithm (https://github.com/dejunlin/hicrep, v0.2.6), with the smoothing factor and the maximum genomic distance set to 3 and 5Mb, respectively. The weighted average of SCCs from individual chromosomes (using chromosome lengths as the weights) were reported as the final SCC score for each comparison. Both compartments and TADs were estimated using cooltools (https://pypi.org/project/cooltools/, v0.4.0). For compartments, the eigenvalue decomposition was performed on the 100kb intra-chromosomal contact maps, and the first eigenvector (PC1) was used to capture the “plaid” contact

pattern. The original PC1 was oriented according to H3K4me3 ChIP-Seq tracks of corresponding cells, so that positive values correspond to active genomic regions and negative values correspond to inactive regions. For TADs, genome-wide insulation scores (IS) were calculated at 25kb with the window size setting to 500kb. Finally, the chromatin loops were identified at 5kb resolution using a Python implementation of the HiCCUPS algorithm (https://pypi.org/project/hicpeaks/, v0.3.5).

Supplementary Method S2. ChIP-Seq data sources and processing

As mentioned above, we used H3K4me3 ChIP-Seq tracks to orient the original PC1 so that the positive PC1 values correspond to active regions and negative PC1 values correspond to inactive regions. For IMR90, we downloaded the track from ENCODE (https://www.encodeproject.org/) with accession code ENCFF518GFI, and converted the original coordinates from hg38 to T2T-CHM13 using CrossMap (v0.5.2) (Zhao, et al., 2014) . For CH12-LX, we downloaded the track from ENCODE with accession code ENCFF012DBS, and converted the coordinates from mm10 to mm39. For zebrafish muscle, we downloaded the raw sequencing reads from the SRA database with accession code SRR9662073, mapped the reads to danRer11 using BWA-MEM (v0.7.17), and generated the signal track using "macs2 callpeak" (v2.2.7.1) with "-B" and "--SPMR" parameters.

Supplementary Method S3. Overview of the HiCLift framework

The inputs to HiCLift include two parts (Supplementary Fig. S1). The first part is a file containing the chromatin contact information. This file can be either a pairs file with each row representing a pair of interacting genomic loci in base-pair resolution, or a matrix file, which stores interaction frequencies between genomic intervals of fixed size. The second part is a UCSC chain file, which describes pairwise alignment that allows gaps in both assemblies simultaneously. Internally, HiCLift uses interval trees to efficiently search for a specific genomic position in a chain file and locate the matched position in the target genome. The converted chromatin contacts will be reported in either a sorted 4DN pairs file, which can be directly used to generate contact matrix in various formats, or a matrix file in .cool or .hic formats.

Supplementary Method S4. Coordinate conversion for contact pairs

HiCLift supports two kind of pairs files: the pairs format defined by the 4D Nucleome Data Coordination and Integration Center (DCIC) (https://github.com/4dn-dcic/pairix/blob/master/pairs_format_specification.md) and allValidPairs defined by HiC-Pro (https://nservant.github.io/HiC-Pro/RESULTS.html). Both formats define contact pairs in base-pair resolution, with each row representing genomic coordinates of a pair of interacting genomic loci. HiCLift iterates each row of a pairs file, searches for the coordinates in the IntervalTree constructed from the

input chain file, and maps to the target genome. A pair of loci is retained only if both sides can be uniquely mapped to the target genome. The input pairs file can be plain text file, gzip/bgzip compressed file (.gz) or lz4 compressed file (.lz4).

Supplementary Method S5. Coordinate conversion for contact matrices

Suppose there is a contact matrix $\boldsymbol{M}$, where each value in the matrix M_{ij} represents the contact frequency/count between bin i and bin j. At a given resolution, each bin represents a genomic interval of fixed size. Therefore, the precise interacting loci for each contact are unknown in such a matrix. To maximize the mappability ratio, for each pair of bins, HiCLift searches for loci that can be uniquely mapped to the target genome, and randomly samples a pair of mappable loci for each contact between corresponding bins.

Two matrix formats are supported: cool (Abdennur and Mirny, 2020) and hic (Durand, et al., 2016). Both are official data formats for the 4D Nucleome consortium. For the hic format, since multiple matrices at various resolutions are stored in a single file, HiCLift automatically detects and reads data from the one at the highest resolution.

Supplementary Method S6. Description and source of chain files

HiCLift uses the UCSC chain file (https://genome.ucsc.edu/goldenPath/help/chain.html) to perform coordinate conversion between two genome assemblies. The chain file represents pairwise alignments between two assemblies as a series of “chains”, with each chain starting with a “chain header” line that specifies the coordinates and orientation of the aligned regions in each assembly. Following the “chain header” are “chain blocks”, which represent the individual aligned segments within each chain. And each block specifies the size of the ungapped alignment and the number of “gap” characters (representing insertions and deletions) between the end of the current block and the beginning of the next block. Here is an example “chain”:

```
chain 255 chr1 248956422 + 260873 267915 chr1 248387328 + 5618 12693 0
246   0   1
281   0   40
1283  0   1
66    2   0
133   5   0
38    0   1
369   2   0
420   0   1
873   1   0
```

38	*1*	*0*
102	*3*	*0*
3053	*0*	*3*
126		

In this study, the chain file "grch38-chm13v2.chain" for mapping coordinates from hg38 to T2T-CHM13 cat be found at https://github.com/XiaoTaoWang/HiCLift/tree/master/chain-files. Other chain files used in this study were downloaded from https://hgdownload.soe.ucsc.edu/downloads.html.

Supplementary Method S7. Construction of interval trees from a chain file

For clarity, HiCLift uses a chain file to map genomic coordinates from the "original" genome to the "target" genome. For example, in the case of the "grch38-chm13v2.chain" file mentioned above, the hg38 is the original genome, and the CHM13 is the target genome.

An interval tree is a data structure used to efficiently search and retrieve intervals that overlap with a given interval or point. HiCLift internally uses an interval tree implementation from the "kerneltree" library (https://github.com/biocore-ntnu/kerneltree) to construct interval trees from a chain file. For each chromosome of the original genome assembly in the input chain file, HiCLift constructs one interval tree. Each node in the tree represents one "block" of a "chain", and is represented by a tuple containing the start and end positions of the interval on the original genome, as well as an index number that records the order in which each interval is added to the tree. HiCLift also constructs a Python list in line with the interval tree, and appends the "block" information on the target genome each time a node is added to the tree. By combing the interval trees and this Python list, HiCLift is able to quickly search for a specific genomic position in the input chain file and then use the index number to locate the matched position in the target genome.

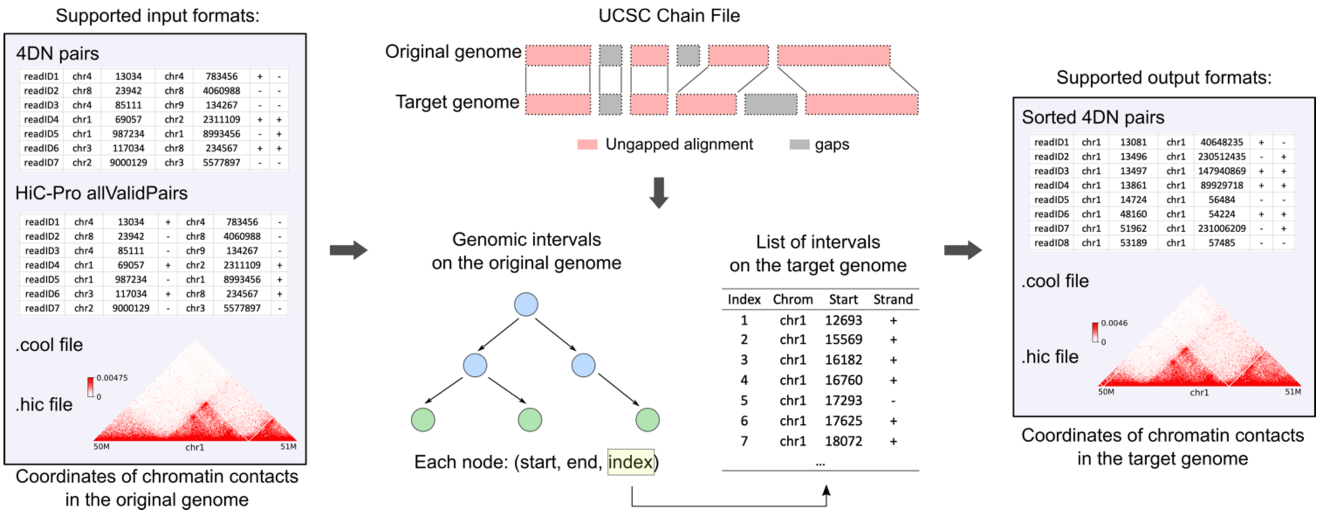

Supplementary Figure S1. The overall design of HiCLift.

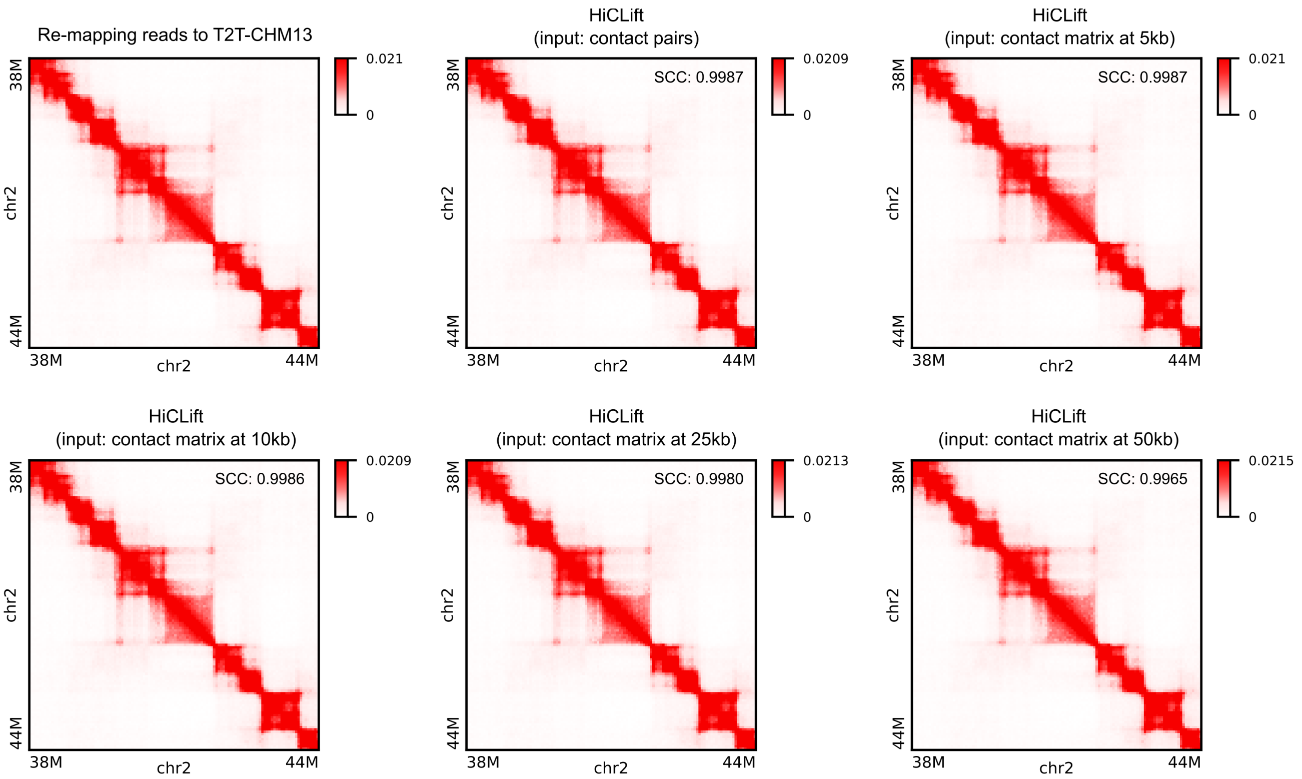

Supplementary Figure S2. Comparison of Hi-C contact maps at 50kb resolution derived from read re-mapping and HiCLift on the IMR90 dataset. We ran HiCLift with contact pairs, and contact matrices at different resolutions as input to convert contact coordinates from hg38 to T2T-CHM13. The results are highly similar to the one with reads re-mapped to T2T-CHM13 regardless of the input data format and resolution. The stratum-adjusted correlation coefficient (SCC) between HiCLift and read re-mapping is indicated in each case.

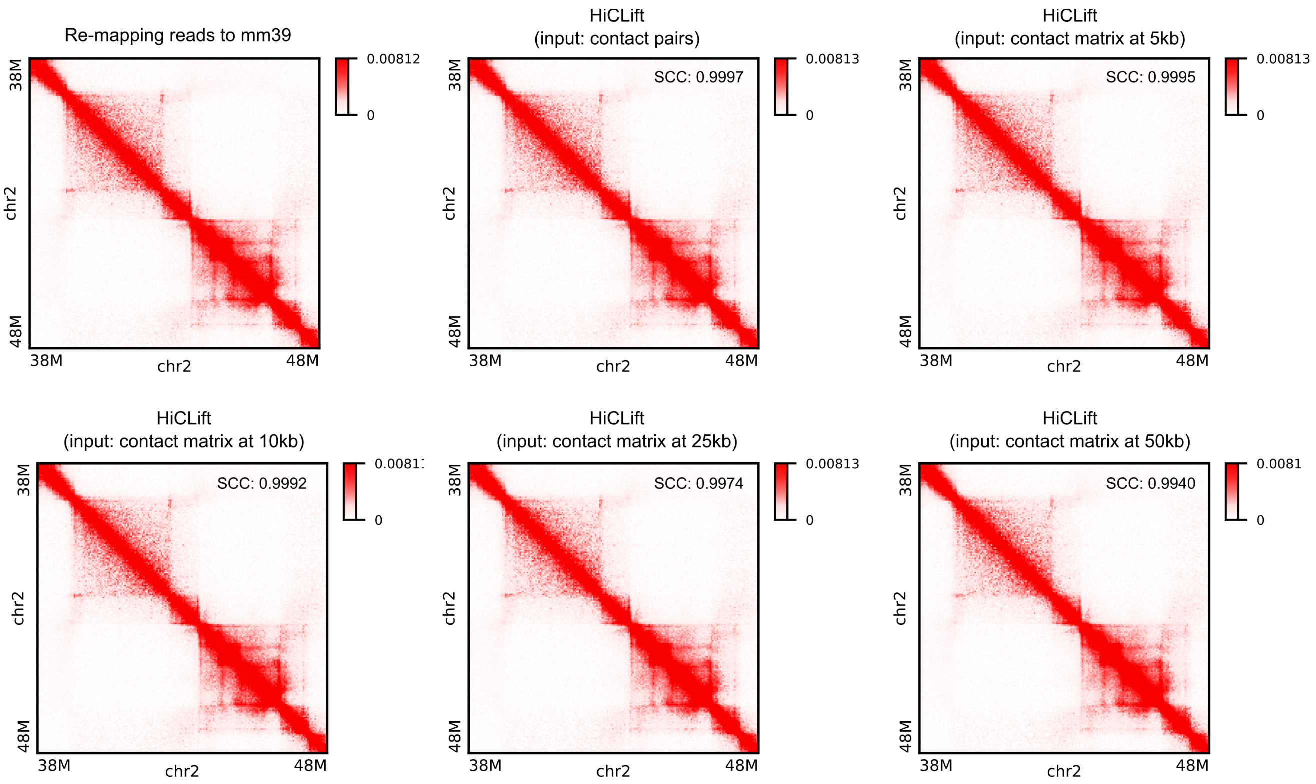

Supplementary Figure S3. Comparison of Hi-C contact maps at 50kb resolution derived from read remapping and HiCLift on the CH12-LX dataset. We ran HiCLift with contact pairs, and contact matrices at different resolutions as input to convert contact coordinates from mm10 to mm39. The results are highly similar to the one with reads re-mapped to mm39 regardless of the input data format and resolution. The stratum-adjusted correlation coefficient (SCC) between HiCLift and read re-mapping is indicated in each case.

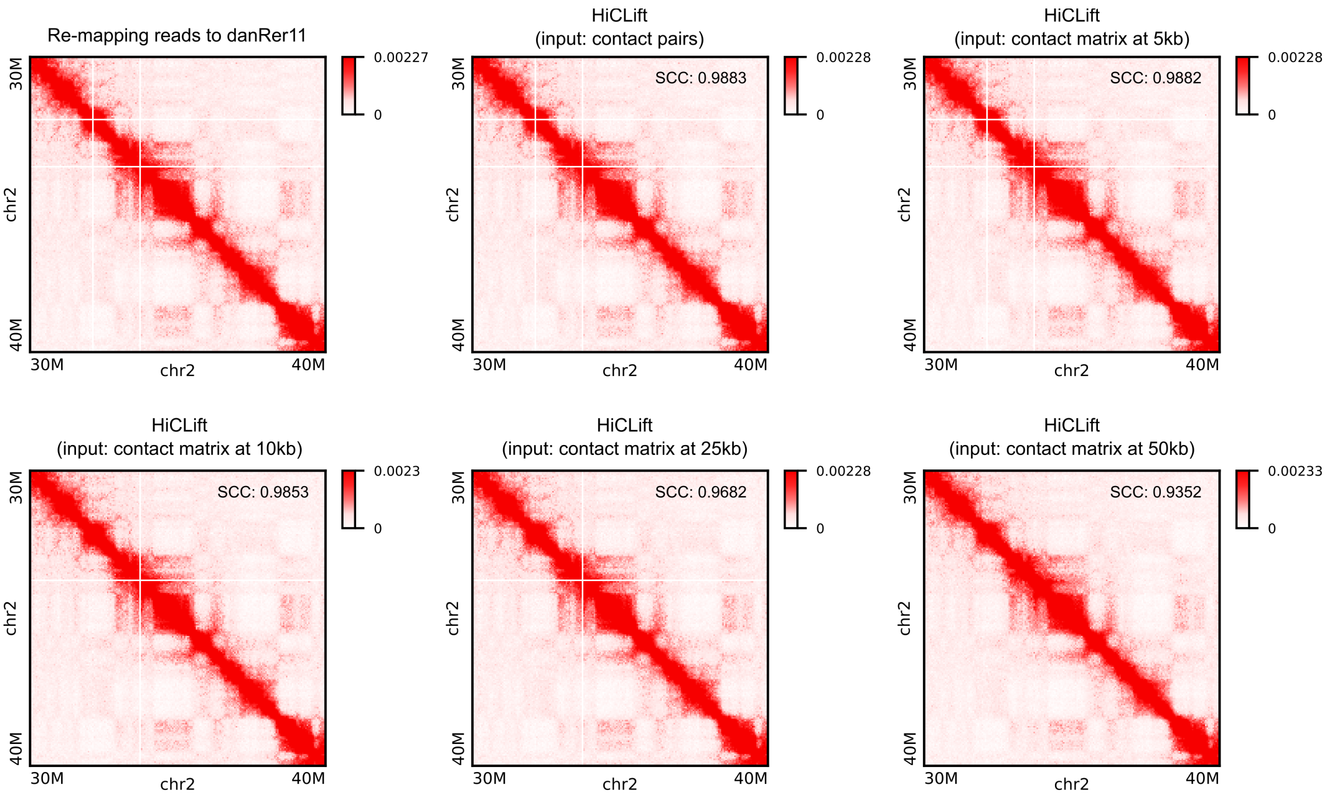

Supplementary Figure S4. Comparison of Hi-C contact maps at 50kb resolution derived from read remapping and HiCLift on the zebrafish dataset. We ran HiCLift with contact pairs, and contact matrices at different resolutions as input to convert contact coordinates from danRer10 to danRer11. The results are highly similar to the one with reads re-mapped to danRer11 regardless of the input data format and resolution. The stratum-adjusted correlation coefficient (SCC) between HiCLift and read re-mapping is indicated in each case.

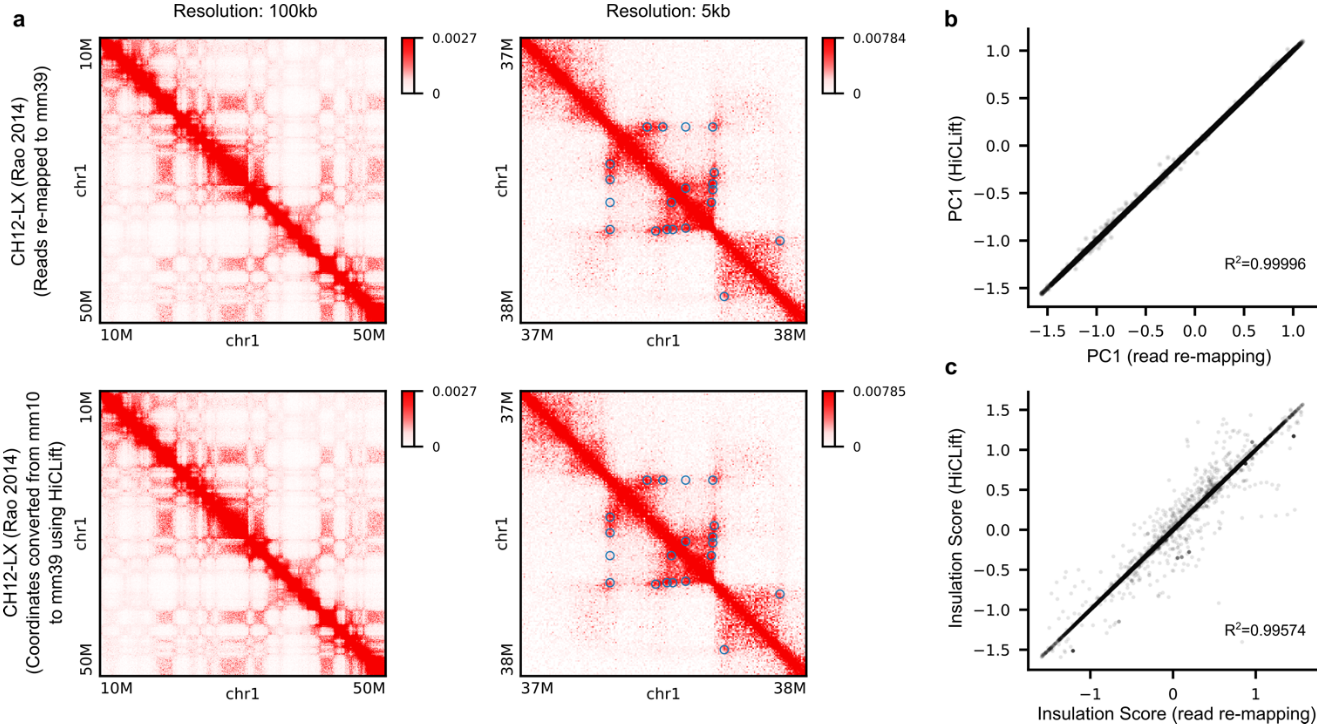

Supplementary Figure S5. Accuracy of HiCLift on a mouse Hi-C dataset (Rao 2014, CH12-LX). **(a)** Example regions comparing contact matrices obtained from HiCLift with the matrices from read re-mapping. The blue circles indicate the detected chromatin loops on corresponding maps. **(b)** The first principal component (PC1) for characterizing the chromatin compartment pattern at 100kb resolution is compared between the two methods. **(c)** The insulation scores for capturing chromatin domain boundaries at 25kb resolution are compared between the two methods.

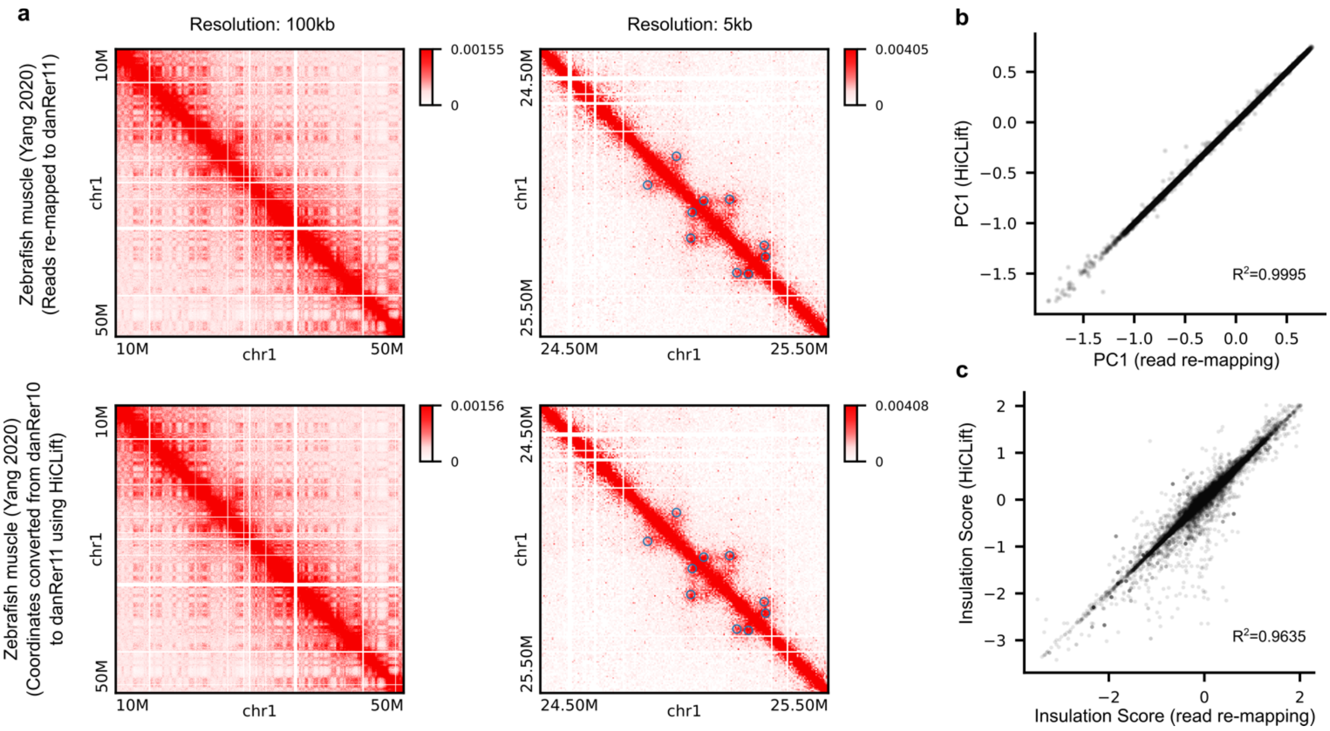

Supplementary Figure S6. Accuracy of HiCLift on a zebrafish Hi-C dataset (Yang 2020, muscle tissue). (a) Example regions comparing contact matrices obtained from HiCLift with the matrices from read re-mapping. The blue circles indicate the detected chromatin loops on corresponding maps. **(b)** The first principal component (PC1) for characterizing the chromatin compartment pattern at 100kb resolution is compared between the two methods. **(c)** The insulation scores for capturing chromatin domain boundaries at 25kb resolution are compared between the two methods.

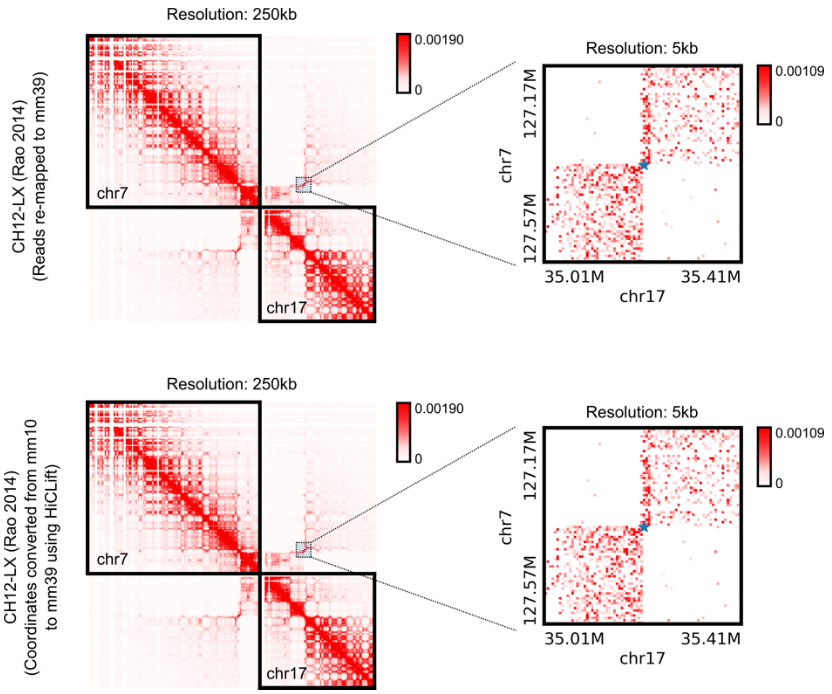

Supplementary Figure S7. Accuracy of HiCLift in converting inter-chromosomal contacts in CH12-LX. (left) The inter-chromosomal contacts show that there is a reciprocal translocation between chr7 and chr17 in the CH12-LX cell line. **(right)** Enlarged Hi-C maps of the highlighted region on the left. The blue stars represent the breakpoint loci of this translocation. The chromatin contacts obtained from HiCLift (second row) in this region are compared with those obtained by re-mapping the raw reads (first row).

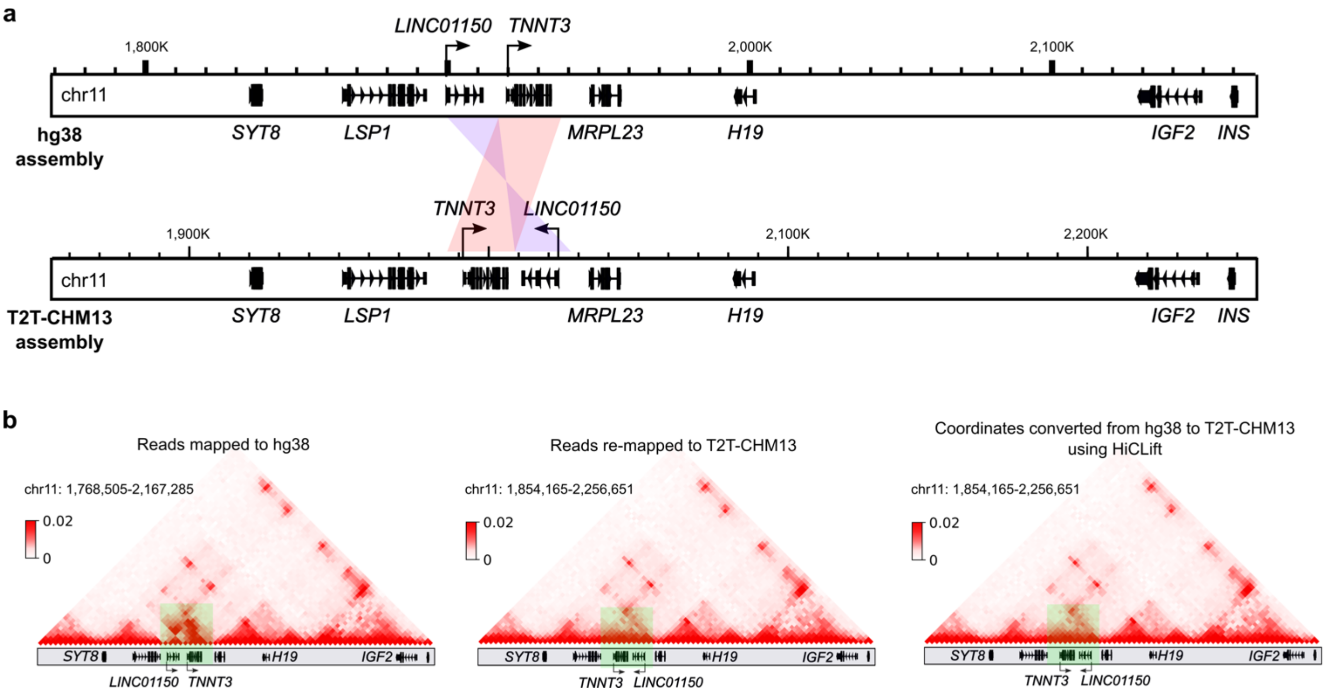

Supplementary Figure S8. Accuracy of HiCLift at inaccurately assembled regions. (a) Comparisons of hg38 and T2T-CHM13 genomes show that the *LINC01150* gene is inverted and inserted upstream of *TNNT3* in the hg38 assembly. **(b).** Comparisons of chromatin contacts (from a Micro-C dataset in H1-ESC cells) mapped to hg38, mapped to T2T-CHM13, and converted from hg38 to T2T-CHM13. The highlighted regions show that abnormal chromatin contacts exist in hg38 due to assembly errors, but are absent from T2T-CHM13.

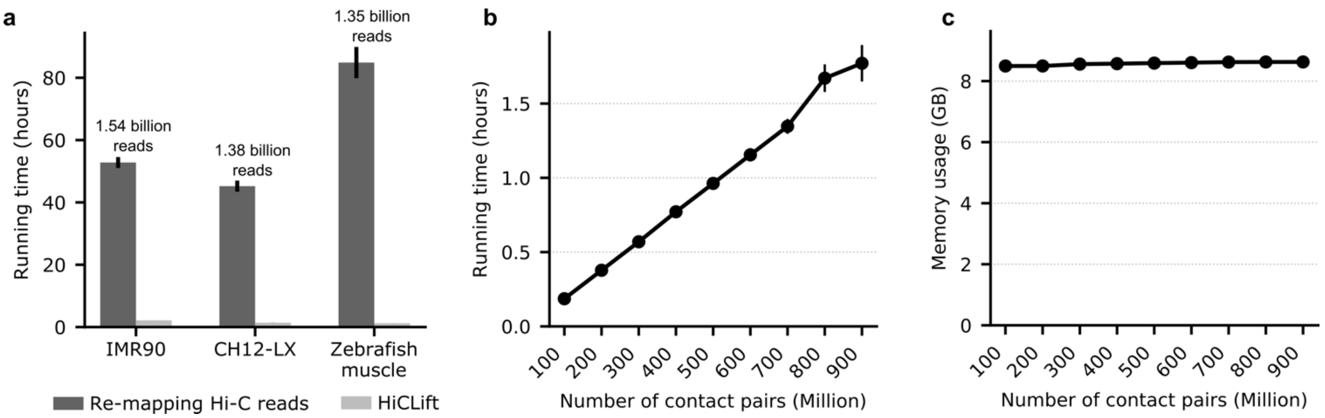

Supplementary Figure S9. Computational efficiency of HiCLift on the benchmark datasets. **(a)** Running time comparison of HiCLift with Hi-C data re-processing on the benchmark datasets. For each dataset and each method, we repeated the computation for 3 times, and the error bars represent the standard deviation for corresponding 3 repeats. **(b-c)** Running time and memory usage of HiCLift on the down-sampled IMR90 datasets. The error bars represent the standard deviation for 5 repeats at each sequencing depth.

References

Abdennur, N. and Mirny, L.A. Cooler: scalable storage for Hi-C data and other genomically labeled arrays. *Bioinformatics* 2020;36(1):311-316.

Akgol Oksuz, B., *et al.* Systematic evaluation of chromosome conformation capture assays. *Nat Methods* 2021;18(9):1046-1055.

Durand, N.C., *et al.* Juicer Provides a One-Click System for Analyzing Loop-Resolution Hi-C Experiments. *Cell Syst* 2016;3(1):95-98.

Rao, S.S., *et al.* A 3D map of the human genome at kilobase resolution reveals principles of chromatin looping. *Cell* 2014;159(7):1665-1680.

Yang, H., *et al.* A map of cis-regulatory elements and 3D genome structures in zebrafish. *Nature* 2020;588(7837):337-343.

Yang, T., *et al.* HiCRep: assessing the reproducibility of Hi-C data using a stratum-adjusted correlation coefficient. *Genome Res* 2017;27(11):1939-1949.

Zhao, H., *et al.* CrossMap: a versatile tool for coordinate conversion between genome assemblies. *Bioinformatics* 2014;30(7):1006-1007.
